# Information sharing through digitalisation in decentralised supply chains

**DOI:** 10.1007/s10479-022-05105-4

**Published:** 2022-12-18

**Authors:** Dimitris Zissis

**Affiliations:** Norwich Business School, University of East Anglia, Norwich, NR4 7TJ UK

**Keywords:** Misinformation, Incentive compatibility, Mediator system, Coordination, Win–win situation, Cloud platform technologies

## Abstract

This study investigates the impact of private information on decision making process and how emerging technologies can facilitate information sharing and reduce misinformation in decentralised settings. Focusing on business environments, we examine if information sharing between distinct partners can be a mutually beneficial option. In principle, information affects the preferences and the actions of decision makers and usually contributes to inefficiencies for the entire system. A supply chain with two rational firms is considered; the firms have conflicting objectives and possess information that cannot be verified. Real-time communication through a cloud platform is allowed, before the firms finalise their strategies. During the communication phase, both firms are free to report whatever information optimises their individual objectives, even fake. Misinformation seems a plausible option, especially in competitive environments, since the firms may take advantages from such behaviour. We demonstrate that sharing the actual information can be beneficial for both, under the implementation of an appropriate mechanism that considers the welfare of the entire chain. Despite the individualistic behaviour of independent decision makers, it is doable to eliminate entirely information asymmetry and misinformation. This happens by including sufficient incentives on a mechanism that induce firms to reveal their information, because it is in their self-interest to do so. The value of information and the expected benefits of the voluntary information sharing are calculated, indicating the potential improvement.

## Introduction

The recent disruptions (COVID-19 pandemic and the Russo-Ukrainian crisis) have laid bare several uncomfortable truths regarding the critical role of information, especially in dynamic environments such as global supply chains (Flynn et al. , 2021). Massive shortages and delays have been observed not only about specialised products, but also for basic/essential products such as pasta, palm oil, etc. An indicative example is from the UK grocery sector, where major retailers faced abnormal queues up to 15,000 customers (Daily Mail , 2021), forcing retailers to set quantity restrictions for certain products (The Guardian , 2020). During turbulent periods, a statement regarding new developments is enough to create chaos and an emotional trigger of public anxiety and panic, as the level of uncertainty is extremely high both for the society and the businesses. An example (except for the stock market) is about the changes in buying behaviour, where the customers fear of the unknown and ask for unusually large quantities of products (Kovacs and Falagara Sigala , 2021). The latter contributes to huge backorders, creating significant operational “issues” and challenges to supply chains (Ivanov , 2020). These operational issues should be addressed as soon as possible, since they put firms’ viability on risk.

The developments in information and communication technologies (Wang et al. , 2021) along with the high internet penetration rate (Xiong et al. , 2021) enable cloud services and blockchain technology as facilitators to easy access to information. The rapid growth of social media platforms (Facebook, Instagram, Twitter, etc.) and cloud platforms (Amazon Web Services, Microsoft’s Azure, Google Cloud Platform, etc.) allows the circulation of information in real time, providing also new features that facilitate bilateral connections between independent decision makers and avoid lengthy negotiations (Constantinides et al. , 2018). It is possible to spread news and disseminate information from almost anywhere in the world, anytime, by just using a mobile device. Significant benefits have been reported by the adoption of emerging technologies in supply chains regarding the information aspect (Pournader et al. , 2020). However, there is a debate whether the material that is shared and the sender are trustworthy or not and how to verify information. During abnormal periods the need of verifying information is more intense since everything can be deemed as true. A characteristic example was during COVID-19 pandemic, where governments across the world announced unprecedented measures such as extensive lockdowns, travel restrictions, etc. to reduce the spread of the virus. Many production lines and business activities were suspended until further notice, making everything to seem possible. Under these unprecedented circumstances, common sense does not always prevail and it is hard to spot what is true or not.

Information is a fundamental and vital element of the world, since almost all the decisions (and the outcomes of these) are based on the available information. Focusing on business environments and decentralised settings, nobody is willing to reveal his/her private information for free; since all the parties seek to use their information as a competitive advantage and secure more profits for themselves (Vosooghidizaji et al. , 2020). Hence, the firms select their decisions, attempting to optimise their individual objectives without considering the global optimum. To expect truthful behaviour and information sharing by all the business partners is a relevant assumption (Karabati and Sayin , 2008); however, it is not always realistic since some parties prefer to lie in an attempt to increase their benefits. Thus, misinformation (and/or deception) is a possible choice, as long as the others cannot verify the information that is shared. Examples of private information (that is hard to get verified) are usually related to cost structures, demand, sales data, etc. In this study, misinformation is allowed and examined; as there are several cases in which business partners anticipate more individual gains if they share fake information.

Our main objective is to investigate the impact of private information and information sharing on the performance of supply chains and the role of technology on that. We consider a decentralised setting with two independent nodes that both have private information regarding their operations and the relative costs. The nodes interact with each other to fulfil customer demand. Regarding the information, it is assumed that each node is the only one who knows its own information, and the other one cannot verify it. Communication is allowed as a means of coordinating their decisions. This may lead to reduced operational costs for both. However, the nodes are not willing to reveal their information and intend to use it as a negotiation lever to secure a better agreement for themselves. We employ the Revelation Principle (Myerson , 1991), a fundamental principle of Mechanism Design, to prove that it can be in the nodes’ self-interest to share honestly their information and achieve reduced individual costs.

In this context, the contribution of our work lies in the following directions: (1) to explore the role of information and quantify the value of it in supply chains; (2) to design a framework based on a cloud platform under which information sharing between distinct business partners (even between competitors) is mutually beneficial; (3) to increase the level of efficiency and mitigate the impact of disruptions in supply chains through private information extraction. The importance of sharing and disseminating true and accurate information is more intense during disruptions, since the majority of supply chains are insufficiently prepared for low-probability, high-impact events and the conditions are fragile.

The rest of the paper is organised as follows: Section 2 briefly reviews the related literature. Section 3 presents the model and the necessary formulation. In Sect. 4, we prove that information sharing can be beneficial for both business partners and quantify the value of information in a decentralised supply chain. Conclusions and potential extensions are discussed in Sect. 5.

## Research background

In today’s dynamic and global business environment, information is one of the most valuable assets. This is also evidenced by several quotes that highlight the importance of information; some examples are: “Information is the oxygen of the modern age” by Ronald Reagan and “Bringing together the right information with the right people will dramatically improve a company’s ability to develop and act on strategic business opportunities” by Bill Gates. Good decisions are impossible without information; so, businesses and organisations are constantly looking for more and better information to support their decision-making processes and achieve their targets (Yan et al. , 2021). A significant portion of their budget is allocated to information and how to use it effectively. The latter is conspicuous based on the increasing number of job openings and courses relative to data analytics and business forecasting. In this work, the research background is concentrated on the following streams: (1) the role of information and (2) how emerging technologies can improve the overall performance, focusing mainly on supply chains.

### Information in supply chains

Supply chains consist of several distinct decision makers (nodes) that have different preferences, objectives, and information (Chopra , 2019). The nodes decide on their actions according to the information that they have available, seeking to increase their individual payoffs. In business environments, companies usually have conflicting objectives and are unwilling to share their information. A thorough review in the Supply Chain Management literature reveals significant efforts relative to information and how business partners and competitors handle it (Vosooghidizaji et al. , 2020).

A major stream is devoted to cases in which all the nodes have access to the same information. However, that assumption is restrictive and does not reflect the supply chain dynamics. Over the last twenty years, several researchers have proposed models to tackle information asymmetries in decentralised settings. Cachon and Fisher (2000), Corbett and de Groote (2000), and Ha (2001) were among the first who examined the role of information in supply chains. Specifically, Cachon and Fisher (2000) investigated how the information impacts on the nodes’ strategies and measured the value of information sharing regarding demand and inventory in a chain with a single supplier and *N* identical retailers. Corbett and de Groote (2000) studied a setting in which the buyer possesses information about the inventory holding cost and is not willing to share it with his supplier. The latter assumes a continuous distribution about buyer’s holding cost, while a quantity discount is employed to influence buyers’ ordering behaviour, resulting in reduced cost for the supplier and the entire chain. Ha (2001) showed that the optimal solution for a chain is not feasible under information asymmetries and employed a cutoff policy to improve chain performance.

Opportunities for mutual benefits can arise if the nodes reveal their information (Fiala , 2005). In line with that, Karabati and Sayin (2008) proposed vertical information sharing, seeking to achieve better individual gains for all; but it was assumed that the nodes share honestly their information. However, the main challenge is how to convince individual companies to share their information. In case that only one party has information, there are models to extract it by him/her. A possible way is through the design and the implementation of a screening mechanism that induces the party with information to reveal it, because it is in his/her self-interest (Cakanyildirim et al. , 2012). This is usually not free of charge, as the one who designs the mechanism should pay “information rent” to the other (Zhou et al. , 2019). Nevertheless, there are studies in which a supply chain can achieve the global optimum through such mechanisms (Schoenmeyr and Graves , 2022). There are also works that tackle settings in which the information asymmetry is two-dimensional; e.g. Pishchulov and Richter (2016) considered that both the holding and the setup cost is unknown and proposed an incentive compatible contract to coordinate the chain. The impact of information sharing has also been studied by Inderfurth et al. (2013) who conducted a laboratory experiment and demonstrated that information sharing mitigates inefficiencies.

The situation becomes really complex when all the parties have private information, since the assumption who is responsible to design a screening mechanism is not plausible. Such settings are common in the Economics literature; models based on bargaining games and auctions have been proposed to address the multi-way information asymmetry (Shneyerov and Wong , 2010). Studies on supply chains that examine bilateral information asymmetry are sparse. To the best of our knowledge, the only related work is the one by Zissis et al. (2020) who assumed bilateral information asymmetry in the context of subsidiaries within a group and introduced the notion of a mediator who facilitates group coordination. Shen et al. (2019) provided a comprehensive review about information in supply chains.

This work focuses how to tackle bilateral information asymmetry in a decentralised supply chain. The nodes are not obliged to reveal their private information, as they are independent and rational decision makers. With the advancement of technology, we seek to extract nodes’ private information based on a voluntary basis to enable coordination in decentralised settings.

### Misinformation and emerging technologies

Business entities have information about their cost structures (e.g., operating under overtime shifts, outsourcing some activities, etc.), demand (e.g., different forecasting methods), and/or sales data. Such kind of information is hard to be verified; hence, only the party who has the information knows about it. Business entities are usually distinct decision makers (independent companies); so, they are not obliged to reveal their information. In some cases, they prefer to share incorrect information, as this is beneficial for them. Therefore, misinformation and/or deception is a possible choice and should be considered when we examine and analyse how the companies decide on their actions.

A well-known effect that demonstrates the consequences of making decision based on inaccurate information in supply chains is the Bullwhip Effect (Lee et al. , 1997). That effect is related to information distortion as we move up the supply chain, because every node orders more items than needed at a given time. This contributes to a significantly larger production without being necessary and leads to massive shortages, delays, and increased operational costs across the chain. Information asymmetry is recognised as one of the most powerful sources of the Bullwhip Effect. The reader is referred to Wang and Disney (2016) for a detailed overview regarding the Bullwhip Effect.

The importance of mitigating the information distortion is underlined both by academics and practitioners who seek with the development of technology to propose applicable and realistic solutions (Papadopoulos et al. , 2017; Sheel and Nath , 2019). Technologies enable a spurred tremendous progress in many sectors of the economy (Xu et al. , 2018), allowing the creation of additional benefits. The role of emerging technologies in the Fourth Industrial Revolution is also highlighted as they provide a tool to address effectively operational issues, especially during disruptions (Silbermayr and Minner , 2016; Snyder et al. , 2016).

Focusing on information, technology can play a catalytic role in managing dissemination of it by mitigating the impact of inaccurate information/fake news (Tandoc et al. , 2018) and increasing the performance of the entire chain. A recent study has revealed that collaborations among independent companies can be possible through the implementation of innovative technologies (Cisneros-Cabrera et al. , 2021), creating benefits for all the participants. This indicates room for improvement in business environments among partners or even competitors. Nowadays, there is an increasing trend of digital platforms to facilitate collaboration and information sharing among business partners (Constantinides et al. , 2018). Furthermore, several studies have been flourished that adopt emerging technologies (Akter et al. , 2022); such as Blockchain Technology (Babich and Hilary , 2020; Pournader et al. , 2020) and Artificial Intelligence (Baryannis et al. , 2019) to address operational issues and promote sustainability in supply chains (Tsolakis et al. , 2022). We refer the reader to Yang et al. (2021) about the drivers, processes, and impact of the adoption of digital and emerging technologies in supply chains; as, a detailed review of that area is out of the scope of this study.

The main idea of this work is to use a cloud platform and support an external entity to design an incentive compatible mechanism, seeking to extract nodes’ private information and optimise the welfare of the entire chain. Hence, supply chain coordination can be possible in decentralised settings with decision makers who behave selfish. The cloud technology platform enables features that facilitate the design and the implementation of such mechanisms in business environments.

## Analytical formulation

### Model description

We consider a decentralised supply chain with two nodes, a supplier and a retailer, which trade a single product. We denote the supplier by *S*, the retailer by *R*, and refer to them using female and male pronouns, respectively. The nodes interact with each other to satisfy customers’ demand *D* ($D>0$); since there are no other ways to fulfil demand. Demand *D* is referred to a specific period. The supplier makes goods available to the retailer who is responsible to satisfy customers’ demand on time. Shortages and backorders are not allowed, since the demand is assumed deterministic and known to both nodes (Sucky , 2006). We assume that nodes have agreed on the wholesale price; i.e., the price that the retailer pays to the supplier per unit of product. Both nodes are rational, risk neutral, and have private information that affects how they decide on their actions. Specifically, the nodes select their decisions under the objective to minimise their own cost, based on the available information that they possess.

The order quantity *Q* ($Q > 0$) is selected by the retailer who has the market power and interacts with the (end-)customers. Thus, he places an order to the supplier who starts the production and prepares the exact number of goods. The supplier cannot keep finished goods as inventory due to limited storage capacity at her premises and completed orders (equal to the order quantity) are directly forwarded to the retailer. The supplier operates under a lot-for-lot policy, paying a setup cost per order and a production cost per unit, denoted by $K_S$ ($K_S > 0$) and $P_S$ ($P_S > 0$), respectively. To clarify that the supplier does not incur any inventory holding costs, as she keeps the finished goods until the production batch to be completed; a negligibly period of time as the production rate is assumed to be large. The setup cost is fixed (independent of the order quantity) and known to both nodes. However, the production cost is known only to the supplier. Given that the wholesale price is agreed and the customers’ demand is deterministic, we omit the fixed term regarding the value of selling the products to the retailer; as it does not affect the shape of her cost function. Based on the above, the supplier’s cost for a period is expressed as:

1 $$\begin{aligned} C_{S}(Q) = K_{S}D/Q + P_SD. \end{aligned}$$

The retailer who is responsible to decide on the order quantity, pays ordering and holding cost. The holding cost of keeping one unit as inventory for a period (same period with the demand *D*), $H_R$ ($H_R > 0$), is assumed to be a percentage of the unit production cost (Krajewski et al. , 2019). To highlight that the holding cost is linked with the production cost (not with the wholesale price as is usually assumed in the literature); we assume that, as the retailer’s holding cost may be affected by the production mode (factory, process, raw material, etc.). The ordering cost is fixed (independent of the order quantity), known to both nodes and is denoted by $K_R$ ($K_R > 0$). Similarly as above, we omit the payment regarding the wholesale price. Thus, the retailer’s cost function for a period is expressed as:

2 $$\begin{aligned} C_{R}(Q) = K_{R}D/Q + H_{R}P_{S}Q/2. \end{aligned}$$

We observe that the supplier’s cost is solely a function of the order quantity *Q* (retailer’s decision). If the supplier could decide about it, she would favor as large as possible quantities since her cost (Eq. 1) is a decreasing function of *Q*. The total supply chain cost is equal to the sum of supplier’s and retailer’s cost (Eqs. 1 and 2), denoted by $C_{J}(\cdot )$. It can be easily observed that the optimal order quantity for the chain, $Q^J$ (i.e., the centralised solution) is larger than the optimal retailer’s order quantity, $Q^R$ (i.e., the one that minimises retailer’s cost function - decentralised solution). The calculations of the optimal order quantities are provided in the appendix. Since the retailer decides on the order quantity, the total cost is not the optimum; indicating room for improvement.

A higher order quantity is preferable from both the chain and the supplier’s perspective. However, that results in increased cost for the retailer, making him not willing to accept it; unless he receives sufficient incentives that cover his additional cost. In this work, a transfer payment is employed to redistribute the total cost on both nodes and facilitate the coordination. In that sense, we allow the supplier to provide a quantity discount to the retailer and share some of her potential benefits with him, as an incentive to induce him to increase the order quantity. Thus, the supplier is a decision maker who selects the quantity discount that will be offered to the retailer. In case of complete information, there are applicable ways such as quantity discounts, two-part tariffs, rebates, etc., under which the nodes coordinate their decisions (Arshinder et al. , 2008). However, the most challenging area relating to coordination is for incomplete information settings, where the coordination is hard to achieve and in some cases is not even possible (Zissis et al. , 2015).

### Private information

Both nodes have private information; the supplier about the production cost and the retailer about the holding cost. Specifically, the information asymmetry is modelled by assuming that the supplier’s production cost and the retailer’s holding cost are discrete random variables. Our aim is to examine whether it can be beneficial for distinct decision makers to share honestly their information, creating additional benefits. Note that, they are not obliged to do it, as they are independent entities with conflicting objectives. They make decisions seeking to optimise their individual costs. Hence, misinformation should be considered as a possible choice, which is a relevant assumption as long as the others cannot verify the information that is shared.

To model private information and examine misinformation, we employ a Bayesian game (Gibbons , 1992); a common approach in supply chain analysis (Cachon and Netessine , 2006). Thus, a finite set is used to model private information. Every element of the set incorporates all the information that a node can have. Obviously, every node has a different set, reflecting his/her information. Each time only one element of the set is selected, which is known only to the corresponding node. The selected element (denoted by *t*) includes all the private information that a node possesses, and we refer to that node as to be type-*t*. Every node is free to act as any of his/her possible types attempting to optimise his/her individual objectives. In case that a node does not act according to his/her actual type, this means that he/she selects the option of misinformation. The assumption of a finite set, regarding the information that a node has, is more realistic for practical problems (Lovejoy , 2006); as (1) the prices and business decisions are discrete, and (2) the possible alternatives are limited.

To drive the research process in a realistic setting, we consider that the single product manufactured (by a supplier) is based on raw materials that can be sourced to alternative providers. We adopt a dual sourcing strategy by considering two alternative options for her. Specifically, the production cost per unit ($P_S$) can be either cheap $P_c$ with probability *q* or expensive $P_e$ with probability $1-q$ (probability of failure of the cheap provider). A dual sourcing strategy is a common approach, after the March 2000 fire at Philips microchip plant in Albuquerque, that led two of the cell-phone giants (Nokia and Ericsson) to chaos (Yu et al. , 2009). The providers offer different prices for such materials and have discrete and limited capacities, known to both nodes. However, the limited capacities do not allow the a priori assumption of “lower price selection” (Stevenson, 2015, Ch.5). The supplier learns if the cheap provider is able to deliver the raw materials or she needs to use the expensive one; i.e., she knows the real value of $P_S$ before deciding on the discount. The retailer does not have access to that information; so, he considers $P_S$ as a discrete random variable such that: $P(P_{S}=P_{c})=q=1-P(P_{S}=P_{e})$.

Regarding the retailer’s holding cost, it is assumed that there are two alternatives. The retailer keeps inventory at his owned warehouse with a low cost per unit, $H_l$, which happens with probability *p*. In case that his warehouse is running out capacity (with probability $1-p$), he operates through an outsourcing storage facility; however, that option incurs a higher holding cost per unit, $H_h$. Hence, the actual cost depends on which facility will be used. The retailer knows if his facility is available or not, before deciding on the order quantity. The supplier is not aware of which facility will be used; so, she considers $H_R$ as a discrete random variable such that: $P(H_R=H_{l})=p=1-P(H_R=H_{h})$.

In the proposed setting both nodes have discrete private information; each has two alternatives. Hence, there are four possible combinations regarding the information (nodes’ type). The availability of the raw materials is not related with the storage facilities; so, the probabilities of all the combinations (“chain profiles”) are calculated by multiplying the probability of the first event by the second. These are presented in Table 1.

**Table 1:** Probabilities for the possible “chain profiles”

Raw material	Warehouse	Probability
Expensive	Owned	$(1-q)p$
Expensive	Outsourcing	$$(1-q)(1-p) $$
Cheap	Owned	*qp*
Cheap	Outsourcing	$q(1-p)$

The Revelation Principle is used to prove that it can be beneficial for both nodes to share their information. For the implementation of the Revelation Principle, the reservation levels of nodes are required. The reservation level is defined as the cost that a decision maker pays under the worst-case scenario for him/her (Gibbons , 1992). In other words, the reservation level reflects on the maximum cost that a node may shoulder. That cost depends on the information that a node has, which is a relevant assumption in business environments as different information leads to different managerial decisions and related costs (Cakanyildirim et al. , 2012). Hence, the reservation level is not a single value, but it takes different values (as many as the alternative options for each node). In this work, it means that there are two values about the reservation level of each node. Specifically, the worst case scenario for the retailer is when the production cost is expensive and discounts are not provided. So, he decides on the order quantity minimising his cost function (Eq. 2), given he knows which storage facility will be used. Thus, his reservation level is:

3 $$\begin{aligned} \text {Retailer:} {\left\{ \begin{array}{ll} C_{R,l}^+= \sqrt{2K_{R} DH_{l} P_{e}}, &{}\text {if owned warehouse is used,}\\ C_{R,h}^+= \sqrt{2K_{R} DH_{h} P_{e}}, &{}\text {otherwise}. \end{array}\right. } \end{aligned}$$

For the supplier, the worst case scenario is when she receives a small order quantity (as her cost is a decreasing function of *Q*). The minimum order that she has to deliver is when the retailer behaves as to operate under the expensive storage facility, assuming that the production cost is expensive as well. In that case, the minimum order quantity equals to $\sqrt{2K_{R}D/ H_{h} P_{e}}$. However, the supplier knows the actual production cost (i.e., if the cheap provider is available or not); so, her reservation level is:

4 $$\begin{aligned} \text {Supplier: } {\left\{ \begin{array}{ll} C_{S,c}^+= K_S\sqrt{DH_{h}P_{e}/2K_{R}}+P_{c}D, &{}\text {if cheap provider is used,}\\ C_{S,e}^+= K_S\sqrt{DH_{h}P_{e}/2K_{R}}+P_{e}D, &{}\text {otherwise}.\\ \end{array}\right. } \end{aligned}$$

### Information sharing

In this context, exchange of information is a critical factor to the chain performance. To investigate the role and the value of information, the nodes are allowed to communicate any information they possess through a mediation system. The idea behind the communication is to coordinate their decisions and achieve the minimum cost for the chain. This creates extra benefits that may lead to reduced individual costs for both. The communication occurs before the nodes finalise their decisions and due to technology developments is effective in real time. During the communication phase, the nodes are free either to share any information (real or fake) or to decline the communication. They are willing to take part to communication if that is aligned with their individual objectives and their reservation levels are not violated.

**Fig. 1:**
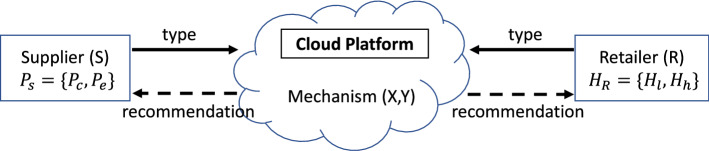
Work overview

The mediation system is considered credible by promoting the welfare of the entire chain and facilitates the communication in real time, through a cloud platform. The communication is effective in a short period of time, avoiding negotiations between the nodes. The mediation system comprises the design of a mechanism that makes recommendations to the nodes about their decisions (i.e., the quantity discount for the supplier and the order quantity for the retailer) for all the possible chain profiles. Specifically, at the beginning of the communication phase, the mediation system releases a mechanism that defines nodes’ decisions for all the possible chain profiles and then asks from both nodes to report confidentially their information. Every node reports his/her type by using the platform, while is unaware of the reported type by the other node. Due to the emerging technologies that exist today, the mediator system is informed immediately while each node does not have access on the reported type of the other. After collecting the reported information, the mediation system makes recommendations about the nodes’ decisions, according to the announced mechanism. To highlight that every node learns only the recommendation that is designed for him/her at that stage; a necessary feature of the coordination mechanism. A cloud platform allows this “restricted” access to information for every node. Figure 1 depicts nodes’ interactions with the cloud platform.

The nodes are not obliged to take part in the communication and share their private information. In terms of modelling, this means that both nodes are informed about the mechanism and the related recommendations, and then select whether they join to the communication by reporting real or fake information. Therefore, the mechanism should include incentives to attract both nodes to participate. The incentives that ensure voluntary participation of the nodes are known in the literature as individual rationality or participation constraints (Myerson , 1991). However, these constraints cannot guarantee that the nodes share honestly their real information.

As the private information cannot be verified and only the nodes have access to that, it may be beneficial for them to share fake information and take advantage of such behaviour. Note that, the nodes are rational decision makers and independent entities; so, a priori truthful behaviour cannot be expected. We anticipate that the nodes may join the communication by reporting fake information in an attempt to achieve better individual gains. This means that misinformation is a possible strategy for them. To make coordination attainable, the mediation system should extract the real information. This is ensured by incorporating incentive compatibility constraints to the mechanism that provide sufficient incentives to the nodes to reveal their information (Myerson , 1991).

The reason of the incorporation appropriate incentives to a mechanism is to induce both nodes for joining the communication and revealing their information on a voluntary basis. The critical aspect of making coordination attainable is to extract actual information from the nodes because it is in their self-interest. It is easy to observe that, in any case, there is always a preferable information that every node would like to share based on his/her cost function (Eqs. 1 and 2 ). Specifically, the retailer prefers a large discount; so, he will always reports high holding cost (even his actual cost is low) unless he receives appreciate incentives to do otherwise. On the other hand, the supplier prefers a large order quantity; so, she always reports low production cost (even the actual cost is high), seeking to induce retailer to increase the order quantity. This occurs as the retailer is not aware of the actual production cost when the deal is discussed. Obviously, he learns the actual production cost when he stores the product, but he cannot amend the financial agreement at that point.

One of the main objectives of this work is to overcome the barrier of information in decentralised settings. Both nodes prefer to report a specific information regardless of the actual one. In the following section, we show how this can be overcome by designing and implementing a mechanism that provides incentives to them to reveal their information (for all the possible chain profiles), because under this strategy their individual objectives are optimised.

## Coordination

In this section, it is demonstrated that the centralised solution can be achieved in a decentralised setting in which the nodes have conflicting objectives and possess private information. This means that independent decision makers are able to capture the maximum level of benefits and improve the performance of the entire chain without any enforcement policy. The achievement of coordination is possible through the introduction of a mediation system that considers and acts according to the objectives of the entire chain, while it overcomes the barrier of private information at the same time. To clarify that the objective of the entire chain is expressed through the cost function $C_{J}(\cdot )$, which is the total chain cost; i.e., the sum of supplier’s (Eq. 1) and retailer’s cost (Eq. 2).

Therefore, the mediation system solves an optimisation problem with objective function to be the $E(C_{J}(Q))$ under participation and incentive compatibility constraints. The philosophy behind this approach is first to increase the joint benefits and reach the maximum level and then to allocate the extra benefits between the participants in a way that reflects their relative powers. Hence, it would be useful to know in advance how much improvement can be achieved through the centralised solution (coordination).

### Benefits from coordination

An interesting question is regarding the potential benefits that can arise, if the nodes coordinate their decisions about the order quantity. In general, the difference between the total cost under the decentralised and the centralised solution is the maximum benefits that can arise from coordination. These benefits will be shared between the nodes and can be also considered as an indication of how much willing the nodes are to achieve coordination. Moreover, it represents the maximum value that a mediator system creates since its integration to the node interactions. This value can be also thought as the magnitude of platform fees that the firms would be prepared to pay and communicate through the platform to coordinate their decisions. In our setting, the potential benefits coincide with the expected value $E(C_{J}(Q^R) - C_{J}(Q^J))$, where: $Q^R$ is the optimal order quantity considering only the retailer’s cost, and $Q^J$ is the optimal order quantity for the entire chain (see appendix). After some algebra, the value $C_{J}(Q^R) - C_{J}(Q^J)$ equals to: $K\sqrt{2DH_{R}P_{S}}$, where $K = (2K_R+K_S - 2\sqrt{K_{R}(K_{R}+K_{S})})/2\sqrt{K_{R}}$. The term $K\sqrt{2D}$ is constant; therefore, the expected benefits $E(C_{J}(Q^R) - C_{J}(Q^J))$ are:

$$\begin{aligned} K\sqrt{2D} \left( p(1-q)\sqrt{H_{l}P_{e}} + pq \sqrt{H_{l}P_{c}} + (1-p)(1-q) \sqrt{H_{h}P_{e}} + (1-p)q \sqrt{H_{h}P_{c}} \right) . \end{aligned}$$

The estimation of the expected benefits that arise from the coordination presupposes that both nodes reveal honestly their information, making possible the selection of the optimal order quantity for the entire chain no matter what chain profile prevails. Hence, the maximum level of the benefits will be available if the mediator system succeed in coordinating nodes’ decisions and overcoming the barrier of private information. An important aspect is that parties are aware of the improvement that can be achieved; i.e., the extra benefits that will be shared between them if coordinate their decisions about the order quantity.

However, the challenges are: (1) whether coordination can be attainable in decentralised settings without restricting participants’ freedom when they select their actions/strategies; and (2) if a mediator system is capable to extract the private information that both nodes have, independent of which chain profile prevails. It is crucial to design a mechanism that obtains the real information for all the chain profiles and not only for some of those. In this work, we focus on the information sharing aspect, examining if that can be a beneficial option for both nodes for every possible combination regarding their private information.

### Existence of coordination mechanisms

One of the main assumptions of this work is that both nodes decide on their actions under the objective to optimise their individual costs, without any consideration of the welfare of the entire chain. This is common in today’s business world which is ultra competitive and every party has different preferences, objectives, and information. Despite the decentralised setting, it is possible for independent firms to reveal their information and achieve coordination (the centralised solution) through the design of an appropriate mechanism. The idea is to implement a mechanism that induce nodes to reveal their information, because it is in their self-interest to do so.

Zissis et al. (2020) has proven a theorem about the existence of coordination mechanisms that incorporate participation and incentive compatibility constraints. In other words, there are mechanisms in which the nodes voluntarily reveal their information because that strategy optimises their individual goals. Thus, individual objectives can be aligned with the entire chain objectives. The outcome of that alignment is to achieve the optimal joint cost and eliminate any inefficiencies. The following corollary is deduced from the theorem that has been proven by Zissis et al. (2020).

#### Corollary 1

There exist (non negative) discounts for all the possible chain profiles that the supplier should provide to the retailer in order to induce him to select the order quantity that minimises the total cost of the chain, because it is in both entities’ self-interest.

A significant result of the coordination mechanisms is the achievement of extracting the private information from both nodes for all the possible chain profiles. This happens by providing sufficient incentives (through a transfer payment—in that work a quantity discount pair is used) to the nodes that align the individual objectives with the objectives of the entire chain. The nodes in an attempt to optimise their costs, coordinate their actions by revealing their information. Hence, information sharing between independent decision makers with conflicting objectives can be a profitable and sustainable strategy for both.

Specifically, an appropriately designed quantity discount pair (*X*, *Y*) is sufficient to make the nodes willing to share honestly their information, reaching a win–win situation. A quantity discount pair (*X*, *Y*) means that the supplier pays a discount *Y* to the retailer, if and only if the order quantity equals to *X*. Based on the Revelation Principle, it is sufficient to consider quantity discounts with four pairs, one for each possible chain profile. The idea is to distinguish the four profiles, inducing the nodes to select the real one through self-selection. The latter is interesting since the private information that the nodes have, cannot be verified. To have access to their information, we should use either enforcement policies or provide sufficient incentives to them to voluntarily reveal it. Attempting to impose actions based on which independent nodes are obliged to share their information cannot be considered as a wise policy, especially in decentralised settings. Thus, the most preferred way in business environments should be the incorporation of sufficient incentives that promote the voluntary information sharing.

Based on Corollary 1, discounts are provided from the supplier to the retailer only for order quantities that coordinate the entire chain and achieve the minimum total cost; these are: $X_{r,s}=\sqrt{2(K_{R}+K_{S})D/H_{r} P_{s}}, r = l, h$ and $s = c, e$. According to theorem by Zissis et al. (2020), the value of the discounts $Y_{r,s}$ are not unique which is a beneficial feature of the coordination mechanisms. That allows to consider secondary objectives (during the design phase) which reflect on other aspects such as the relative power of the nodes and how the extra benefits will be allocated to them in a fair way. That flexibility makes the role of the mediator system significant compared to a facilitator who just helps the parties to coordinate their actions.

Obviously, the minimum values of discounts are preferable for the supplier, while the retailer prefers as large as possible discounts. An interesting observation that arises from the minimum and the maximum values of discounts that achieve coordination, is about the guaranteed benefits that each node captures through the communication and the incorporation of a mediator system. The guaranteed benefits indicate the impact of communication on the nodes’ reservation levels and quantify the value of information. It is out of the scope of this study to allocate the extra benefits to the nodes and make assumptions about the relative power of the nodes. We highlight that the values of the discounts do not affect the benefits that arise from coordination, since the discount that will be implemented is a transfer payment from the supplier to the retailer. The concept of transfer payments is used to redistribute the total cost in a way that provides sufficient incentives to the nodes to reveal their information and streamline the order quantity under which they operate to satisfy the customer’s demand.

## Concluding remarks

Motivated by the recent supply chain disruptions and the related challenges, we have examined the impact of information in decentralised settings and how information sharing can improve the performance of the entire chain. A decentralised setting with two independent nodes (firms) has been considered. Each firm possesses information that is hard to be verified by others, while their preferences are conflicting. Both decide on their actions seeking to optimise their individual objectives, without considering the welfare of the entire chain. They are allowed to communicate about their information and preferences in an attempt to coordinate their decisions and achieve reduced costs. However, they are not obliged to reveal their real information as they are not part of the same group.

Misinformation is a possible option; especially if the firms anticipate to capture more individual benefits by following a such strategy. It is also plausible as every node is aware only of his/her information and there is no way to verify private information. We have shown that information sharing can be always beneficial for firms with conflicting objectives under a mediation system that considers the welfare of the entire chain. The firms are willing to share their information if their objectives are optimised and their reservation levels are not violated. The idea is to design a mechanism that facilitates the communication and includes incentives for both nodes to reveal their information because it is in their self-interest to do so. Therefore, misinformation can be eliminated through a carefully designed mechanism; this results in increased supply chain efficiency, allowing win–win situations.

Nowadays with the developments of the technology, such mechanisms seem easier to be developed than some years ago. An important aspect of the technological advancement is regarding the implementation cost of mechanisms which is reasonable and not prohibitive. In this study, the mechanism that extracts private information is a mediation system on a cloud platform. The high internet penetration rate allows the implementation of cloud services in real time, avoiding lengthy negotiations. Such instruments should have top priority for the policymakers, as these can address effectively issues about profitability, viability, shortages, etc. by enabling agility and resilience in supply chains (Tsolakis et al. , 2021). This is more intense during disruptions, as all the supply chain actors operate under special conditions that harm efficiency.

The expected benefits from the voluntary revealing of private information and the value of information have been quantified; so, the firms are aware about the improvement that can be achieved if they integrate a mediation system and streamline their decisions. The expected benefits are also an indication about the willingness-to-pay threshold that firms can shoulder (in total) to incorporate a mechanism to their interactions. The value of information represents the power that a decision maker has and how much improvement should be added to his/her reservation level when communication is allowed.

Overcoming the barriers to information sharing between business partners even if they are competitors, is the main finding of this study. Through mechanism design and the Revelation Principle, there are applicable ways to induce independent decision makers to achieve the centralised solution, without restricting their freedom. This is attainable as the mechanism includes incentives that align the individual objectives (of all the parties) with the objectives of the entire chain. Thus, the parties select the strategy that optimises their objectives but at the same time this strategy achieves the optimum for the whole chain. This alignment leads also to significant indirect benefits that are difficult to quantify. For example, as the order quantities that are moved between the supplier and the retailer are increased, environmental benefits arise (Zissis et al. , 2018).

An interesting observation is related with the fact that when both nodes have private information, the inefficiencies caused by information asymmetry can be completely eliminated. However, this is not always possible in settings where only one node has information. The intuitive explanation of that is due to the integration of a mediation system (external entity) which considers the welfare of the entire chain and designs a mechanism to achieve the chain optimum. In models with two nodes where only one has information, it is common for the other one to act as a leader and use a screening mechanism trying to extract information and optimising his/her objectives (Schoenmeyr and Graves , 2022). In principle, the individualistic behaviour of the leader during the design of a screening mechanism does not always allow to reach the centralised solution, resulting in inefficiencies for the chain. The main issue is related to information rent that the node without information should pay to the node with information to get access to his/her information.

There are several directions that seem promising for future research. The first one is to consider models in which the parties have more than two alternatives regarding their information, or have two-dimensional private information. It would be great to investigate the role of two-dimensional information and if it is possible to eliminate the asymmetry under such cases. Another interesting extension would be the incorporation of consumers’ behaviour to the model and how extreme behaviours such as panic buying affects nodes’ interactions and the information sharing. Last but not least, to study supply chain models with more than two decision makers from different tiers, considering also aspects such as lead time and constraints regarding replenishment policies.

## Appendix

### Calculation of the optimal retailer’s order quantity: $Q^R$

The optimal order quantity can be derived by taking the first order derivative of the retailer’s cost function (Eq. 2), setting equal to zero and solving with respect to *Q*. After some algebra, the optimal retailer’s order quantity equals to: $Q^R=\sqrt{2K_{R}D/ H_{R} P_{S}}$.

### Calculation of the optimal order quantity for the entire chain: $Q^J$

Similarly as above by considering this time the total cost of the chain which is: $C_{J}(Q) = (K_{R}+K_{S})D/Q + H_{R}P_{S}Q/2 + P_SD$. After some algebra, the optimal order quantity for the entire chain is: $Q^J=\sqrt{2(K_{R}+K_{S})D/ H_{R} P_{S}}$.
